# Cases of Surgery

**Published:** 1845-09

**Authors:** Daniel Brainard

**Affiliations:** Prof. of Surgery in the Rush Medical College, Chicago


### ILLINOIS

### MEDICAL & SURGICAL JOURNAL.

VOL. II. SEPTEMBER, 1845. NO. 6.

*Cases of Surgery.* By DANIEL BRAINARD, M. D., Prof. of Surgery in the Rush Medical College.

*Diseased Testicle, complicated with Hernia—Castration.*—J. R. B., æt. 40 years, applied to me during the month of August, 1845, with a disease of the right testicle, of which he gave the following history: Eight years previously he first noticed enlargement of the organ, which was then slight. Its growth, slow at first, but rapid of late, has been unattended with pain. At present it is pyriform, its apex extending to the inguinal ring, is 5½ inches in length, and 3½ in its transverse diameter. It is hard, not sensible, has various irregularities on its surface, and presents at the lower part a smooth surface slightly fluctuating. There exists upon the same side an oblique inguinal hernia, the contents of which in a great measure recede when the patient is in a recumbent position.

Castration being determined upon, was performed Sept. 1st, with the assistance of Drs. Herrick and Blaney, in the following manner: An incision was made from the ring to the base of the tumor, and carried through the superficial coverings of it. These were then dissected off, but on coming to the spermatic cord, this was found to be covered on its anterior part, and partly surrounded by the hernial sac, which was then carefully laid open. A piece of omentum was found within it, descending upon the anterior surface of the tumor, and extensively adherent to it, requiring the knife for its separation. Pressing this upward, the cord was insulated, and a ligature passed around it, firmly tied, and the division made below it. As considerable hemorrhage still took place from the divided vessels of the omentum, the principal trunks of these, six in number, were ligated, and a part beyond the ligatures removed with the scissors. The remainder was reduced within the abdominal cavity, being retained at the opening by the ligatures. Stitches, adhesive straps and simple dressings were applied, and no unpleasant symptoms occurred. The patient is at present, Sept. 10th, free from danger.

On examination of the tumor, it was found that the inequalities upon its surface were due to the enlarged divisions of the epididymis, the smooth surface below was of the testicle itself, and a small quantity of water in the *tunica vaginalis* occasioned the fluctuation. On laying it open, the entire mass was found entirely disorganized, small masses of tubercular matter being disseminated through it, and the remainder consisting of a reddish, pulpy, semi-fluid substance.

*Remarks.*—The points of most interest in this case are the course pursued with the omentum, and the manner adopted for tying the cord.

The presence of an irreducible hernia complicates the operation of castration in a most serious manner, and the course to be pursued with the contents of the sac is important to be decided. In this case we have adopted the rule of conduct applicable to the operation of strangulated Hernia, where the same state of things often occurs.

Instead of dividing the spermatic cord, and then tying the arteries, we preferred placing the ligature around the whole of it at once. This, although not the usual method, has in its favor security against hemorrhage, facility of performance, and safety; and if these are not sufficient, the authority and practice of Velpeau. It is not attended by any disadvantages.

Chicago, Sept. 10, 1845.

